# Crystal structures of non-oxidative decarboxylases reveal a new mechanism of action with a catalytic dyad and structural twists

**DOI:** 10.1038/s41598-021-82660-z

**Published:** 2021-02-04

**Authors:** Matthias Zeug, Nebojsa Markovic, Cristina V. Iancu, Joanna Tripp, Mislav Oreb, Jun-yong Choe

**Affiliations:** 1 Department of Chemistry, Biochemistry, and Pharmacy, Goethe University Frankfurt, Frankfurt am Main, Germany; 2 Department of Biochemistry and Molecular Biology, The Chicago Medical School, Rosalind Franklin University of Medicine and Science, North Chicago, Illinois USA; 3 Department of Chemistry, East Carolina Diabetes and Obesity Institute, East Carolina University, Greenville, NC USA; 4 Institute of Molecular Biosciences, Faculty of Biological Sciences, Goethe University Frankfurt, Frankfurt am Main, Germany

**Keywords:** X-ray crystallography, Enzyme mechanisms, Enzymes

## Abstract

Hydroxybenzoic acids, like gallic acid and protocatechuic acid, are highly abundant natural compounds. In biotechnology, they serve as critical precursors for various molecules in heterologous production pathways, but a major bottleneck is these acids’ non-oxidative decarboxylation to hydroxybenzenes. Optimizing this step by pathway and enzyme engineering is tedious, partly because of the complicating cofactor dependencies of the commonly used prFMN-dependent decarboxylases. Here, we report the crystal structures (1.5–1.9 Å) of two homologous fungal decarboxylases, AGDC1 from *Arxula adenivorans,* and PPP2 from *Madurella mycetomatis. *Remarkably, both decarboxylases are cofactor independent and are superior to prFMN-dependent decarboxylases when heterologously expressed in *Saccharomyces cerevisiae.* The organization of their active site, together with mutational studies, suggests a novel decarboxylation mechanism that combines acid–base catalysis and transition state stabilization. Both enzymes are trimers, with a central potassium binding site. In each monomer, potassium introduces a local twist in a β-sheet close to the active site, which primes the critical H86-D40 dyad for catalysis. A conserved pair of tryptophans, W35 and W61, acts like a clamp that destabilizes the substrate by twisting its carboxyl group relative to the phenol moiety. These findings reveal AGDC1 and PPP2 as founding members of a so far overlooked group of cofactor independent decarboxylases and suggest strategies to engineer their unique chemistry for a wide variety of biotechnological applications.

## Introduction

Non-oxidative decarboxylases of phenolic compounds enable the biotechnological production of chemicals such as pyrogallol, catechol, styrene, and cis-cis muconic acid from gallic acid, protocatechuic-acid, cinnamic acid, and related precursors^1–4^. These compounds have versatile use in industry and health, e.g., as common additives to consumer care products, biodiesel^5,6^, and food^7^. They also function as building blocks for fine chemicals such as the antibiotic trimethoprim, the muscle relaxant gallamine triethiodide, the insecticide bendiocarb^1^, or novel anti-cancer drugs like arylspiroborates^8^. Additionally, polyphenolic acids have therapeutic properties in the treatment of obesity, diabetes, cancer, and cystic fibrosis^7,9,10^.

Industrially, gallic acid and its derivatives are produced mainly by the chemical degradation of tannins, which is a low-yield, cost-intense, and highly polluting process^11^. Thus, recent approaches have focused on switching to an environmentally friendly and efficient biotechnological approach^4,12^. Although both titers and pathway efficiency increased steadily in recent years^13^, these compounds’ biotechnological production still suffers from low yield and unwanted side reactions. A major bottleneck of these pathways is the decarboxylation step^1,4,14^.

**Figure 1:**
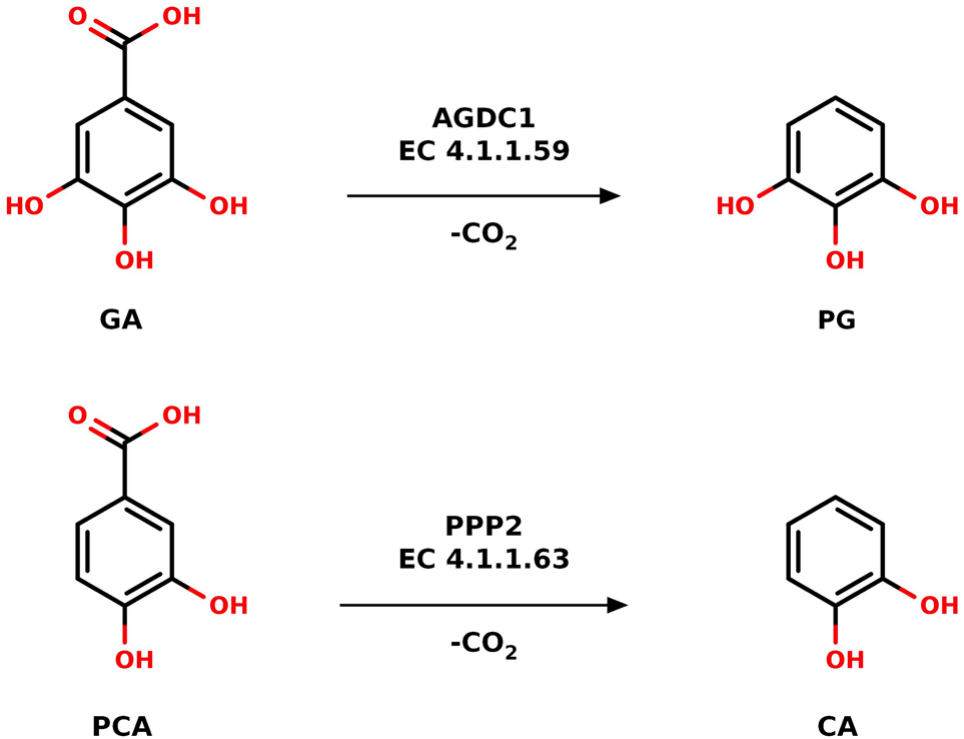
The reactions catalyzed by AGDC1 and PPP2. (Top) AGDC1 facilitates the non-oxidative decarboxylation of gallic acid (GA) to pyrogallol (PG). (Bottom) PPP2 carries out the same reaction for protocatechuic acid (PCA), producing catechol (CA). AGDC1 and PPP2 also act on each other’s substrates but with lower activity. The figure was generated with MarvinSketch (ChemAxon). Enzyme commission numbers were taken from BRENDA (www.brenda-enzymes.org).

The gallic acid decarboxylase AGDC1 was initially isolated from *Arxula adenivorans*^15^. Homologous proteins exist in a variety of lactic bacteria and fungi^16^. Analyses of strain extracts showed that the enzyme is crucial for tannin degradation, catalyzing gallic acid’s decarboxylation to pyrogallol^16^ (Fig. 1). AGDC1 accepts both protocatechuic acid and gallic acid as substrates^16,17^. It acts on both compounds with similar k_cat_ values but has an increased affinity towards gallic acid (K_M_ of 0.7 and 3.2 mM, for gallic acid and protocatechuic acid, respectively)^16^. So, AGDC1 is a promising choice for approaches that seek to overcome the crucial decarboxylation-bottleneck and already demonstrated its potential in yeast-based production platforms. Brückner et al*.*^17^ used AGDC1 in the heterologous production of cis-cis muconic acid in *Saccharomyces cerevisiae*, increasing the yield of the pathway by 22% compared to the bacterial enzyme AroY-C. AroY has been the primary choice for catechol and pyrogallol based pathways^17^. However, its dependence on prenylated flavin mononucleotide (prFMN) requires a stable and balanced cofactor supply, complicating AroY usage in metabolic engineering approaches. Substituting AGDC1 with its close homolog Pea Pathogenity Protein (PPP2) from *Madurella mycetomatis* boosted the titer further by 21%^17^. Remarkably, both AGDC1 and PPP2 do not require an organic cofactor for catalysis.

Hitherto, only a few non-oxidative decarboxylases have been investigated thoroughly because the high oxygen-sensitivity of these enzymes is a significant challenge in protein-purification^11,18^. Based on their substrate specificity and mechanism of action, the so-far characterized non-oxidative decarboxylases are classified into three major groups^19^: decarboxylases of the amidohydrolase superfamily, which feature a divalent metal cofactor; prenylated flavine mononucleotide (prFMN) dependent decarboxylases (UbiD superfamily); and phenolic acid decarboxylases (PADs), which are cofactor independent.

All three classes use a different catalytic mechanism. Decarboxylases of the aminohydrolase family depend mainly on zinc or manganese ions, through which the enzymes act on ortho-substituted phenols. The exact mechanism is debated^20^, but zinc-dependent decarboxylases like ACMSD were reported to employ a zinc-bound hydroxide to generate a tetrahedral intermediate through a nucleophilic attack^21,22^, whereas manganese dependent decarboxylases seem to act in a Kolbe-Schmitt like reaction, chelating both the carboxy- and the ortho-hydroxyl group of the substrate, without the involvement of a water molecule^19,20^. The prFMN cofactor, required for the activity of the UbiD superfamily decarboxylases, was only recently discovered^23^. Evidence obtained from structural^23^ and kinetic^24,25^ studies suggests two distinct modes of action. When prFMN-dependent decarboxylases act on carboxylic acids with conjugated double-bonds, they use a complex 1,3 cycloaddition (Fdc1-type), while those working on aromatic substrates utilize the iminium form of prFMN as an electron sink (AroY-type)^25,26^. Finally, PADs rely solely on acid–base catalysis. However, this requires either ATP hydrolysis to increase phenol acidity or a styrene moiety in the substrate^19^. The unsaturated beta-carbon leads to the formation of a quinone intermediate, which eliminates CO_2_ producing a terminal alkene. AGDC1 and PPP2 fit in none of these categories as both enzymes neither display cofactors bound to their active sites nor require the styrene motif or ATP hydrolysis. Instead, they act on the carboxyl-group directly attached to the aromatic ring^16,17^.

Through their unique mode of action, AGDC1 and PPP2 belong to a structurally and mechanistically uncharacterized class of phenolic acid decarboxylases. These AGDC1-type decarboxylases are well suited for bio-catalysis and white biotechnology, as initial tests revealed an excellent performance compared with their previously used counterparts. These enzymes also offer an interesting case study for a new reaction mechanism. Here, we report the crystal structures of the apo-AGDC1 and the complexes of AGDC1 and PPP2 with the substrate-analog 4-nitrocatechol. Along with functional studies on wild-type and mutant enzymes, these structures allow the first look into the structure–function relationship of these exotic decarboxylases, laying the basis for rational protein-engineering to alter substrate specificity and enhance reaction turnover. Additionally, this report might pave the way to engineer the reverse reaction—the carboxylation of aromatic compounds, which is a crucial and much needed, but challenging step in many modern biocatalysts approaches^27,28^.

## Results

Heterologous recombinant expression of AGDC1 and PPP2, with an N-terminal hexahistidine tag removable by thrombin digestion, was done in *E. coli*. Both enzymes expressed very well and were purified to homogeneity by immobilized metal affinity chromatography, typically yielding 10–20 mg purified protein/liter of cell culture. Enzyme assays and protein crystallization used the tagless, purified protein. Meier *et al*^16^ reported on the kinetic characterization of AGDC1, and we used their studies as a starting point for characterizing the function of AGDC1 and PPP2.

### AGDC1 enzyme activity assay

**Figure 2:**
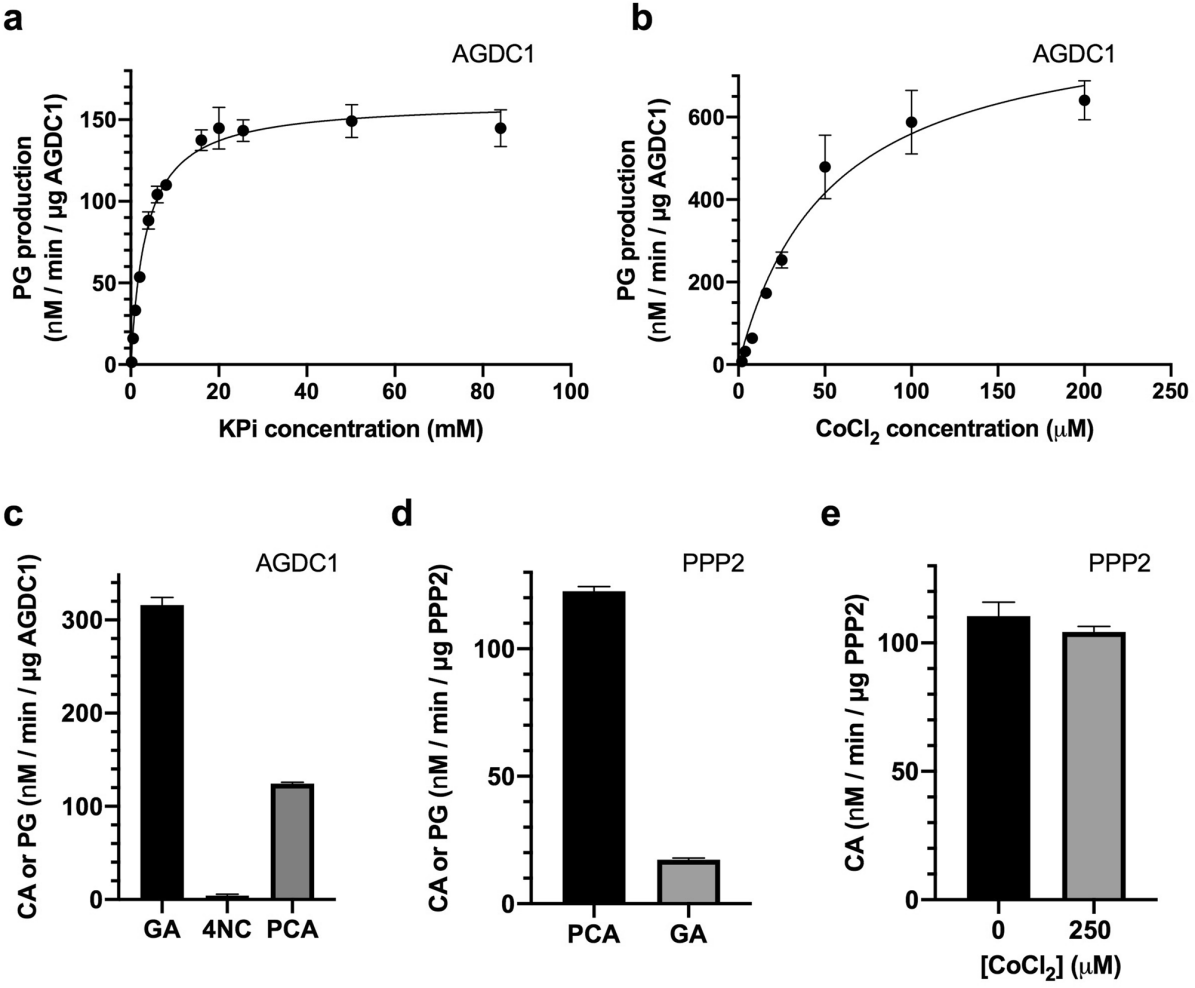
Assay of wild-type purified AGDC1 and PPP2. (**a**) Effect of KPi, pH 6.5, on AGDC1 activity. The assay had 0.5 mM gallate (GA), 50 mM MES, pH 6.5, and 80 µg/ml AGDC1, and was performed at RT. (**b**) Effect of CoCl_2_ on AGDC1 activity. The assay had 0.5 mM GA, 50 mM KPi, pH 6.5, and 40 µg/ml AGDC1, and was performed at 37 ºC. To investigate CoCl_2_ activation, the background activity in the absence of CoCl_2_ was subtracted from the activity in the presence of CoCl_2_. (**c**) AGDC1 activity with 0.5 mM GA or protocatechuic acid (PCA). The inhibition by 0.5 mM of the substrate-analog 4-nitrocatechol (4NC) was checked in the presence of 0.5 mM GA. The assay had 50 mM KPi, pH 6.5, 250 µM CoCl_2_, and 80 µg/ml AGDC1, and was performed at RT. (**d**) Enzyme activity of PPP2 with GA and PCA. The activity assay was performed in the same conditions (50 mM KPi, pH 6.5, 160 µg/ml PPP2), at RT, with 0.5 mM PCA (black) or 0.5 mM GA (grey). (**e**) Effect of CoCl_2_ on PPP2 activity. The assay, performed at RT, had 50 mM KPi, pH 6.5, 0.5 mM PCA, and 160 µg/ml PPP2, with (grey) or without (black) 250 µM CoCl_2_. Consumption of GA was monitored at 259 nm, that of PCA at 250 nm. At the corresponding wavelengths, both PCA and GA had similar absorbance for 0.5 mM, in the same experimental conditions (i.e. same volume, same cuvette, same UV–VIS spectrometer). The addition of protein started the reaction. Error bars represent standard deviation from three independent measurements.

The enzyme activity assay of purified recombinant AGDC1, based on Meier *et al*^16^, monitored the consumption of gallic acid (GA), measured as the decrease of absorbance at 259 nm in time. The cuvette volume (500 µl) and light path (0.2 mm) limited the substrate concentration in the assay to 0.5 mM GA, which was close to its reported K_M_ (0.7 mM)^16^. In 50 mM MES pH 6.5, there was almost no enzyme activity. The addition of KPi pH 6.5, up to 50 mM concentration, increased AGDC1 enzyme activity (Fig. 2a). Half-maximal activation was achieved at a KPi concentration of 3.52 ± 0.33 mM. Therefore, we chose 50 mM KPi, pH 6.5, as the assay buffer, in agreement with earlier findings that indicated a peak of AGDC1 activity in 50 mM KPi buffer at pH 6.2^16^.

We were unable to replicate previous observations related to the effect of EDTA and CoCl_2_^16^, finding instead that EDTA decreased the enzyme activity by 20%, and CoCl_2_ increased enzyme activity by 270% at 37 °C or 40% at room temperature (RT) rather than inhibit it (Fig. 2b, Supplementary Fig. S1). These discrepancies may stem from differences in the protein expression systems. Here, the protein was purified from *E. coli* cell-lysate, whereas previous studies purified the enzyme directly from *A. adenivorans*, possibly resulting in different metal ligation states of the purified enzyme. Half-maximal activation of enzyme activity by CoCl_2_ was achieved at a CoCl_2_ concentration of 54 ± 10 µM (Fig. 2b). Interestingly, all other divalent metals (CaCl_2_, MgCl_2_, MnCl_2_, and ZnCl_2_) tested did not affect or slightly inhibited the enzyme activity, with ZnCl_2_ showing the most potent inhibition (40% activity decrease at 250 µM ZnCl_2_) (Supplementary Fig. S1a), consistent with previous findings^16^.

The canonical activity assay had 0.5 mM GA, 50 mM KPi, pH 6.5, and 250 µM CoCl_2_. In these conditions, on average, the same amount of enzyme had ~ threefold higher activity at 37 °C than at room temperature (RT) (Supplementary Fig. S2). Thus, we used 80 µg/ml AGDC1 for enzyme assay at RT or 40 µg/ml AGDC1 for assay at 37 °C. The addition of 0.5 mM 4-nitrocatechol (4NC) in the canonical activity assay decreased the relative enzyme activity by 98%, indicating that 4NC is an AGDC1 inhibitor (Fig. 2c). When varying the assay buffer (50 mM KPi) pH from 5.0 to 8.0, we found the optimal pH at 6.5—7.0. Co^2+^ activation had the same optimal pH range, with a steep decline to almost no Co^2+^ activation within 0.5 pH units at either side of the optimum pH (Supplementary Fig. S3). As previously reported^16^, AGDC1 was less active on protocatechuic acid (PCA) than on GA. For the same concentration of substrate (0.5 mM) and assay conditions (50 mM KPi, pH 6.5, 250 µM CoCl_2_, 80 µg/ml enzyme), AGDC1 activity with GA was ~ 2.5 -fold higher than with PCA (Fig. 2c). PCA decarboxylation activity monitored the decrease in substrate concentration, measured as the decline in time of the absorbance at 250 nm.

### PPP2 enzyme activity assay

The activity assay of purified PPP2 contained 50 mM KPi, pH 6.5, 0.5 mM PCA, and 160 µg/ml enzyme and was performed at RT. PPP2 activity with PCA was ~ sevenfold higher than with GA in the same assay conditions (Fig. 2d). Unlike with AGDC1, CoCl_2_ did not affect the enzyme activity significantly (Fig. 2e).

### Crystal structure determination

Initial crystals of AGDC1, in space group P2_1_2_1_2_1_, diffracted to 2.0 Å resolution and had an asymmetric unit (AU) with nine protein molecules. Phasing by molecular replacement was not an option since no homologs for AGDC1 had known crystal structures. Multi-wavelength anomalous dispersion (MAD) phasing of crystals for the Selenium-Methionine (Se-Met) derivatized protein or heavy metals derivatives, including Pt, Hg, and lanthanides, failed due to the high number of molecules in AU. Therefore, we tried to determine the crystal structures of AGDC1 homologs to use for molecular replacement phasing. Crystals of an AGDC1 homolog from *Aspergillus niger* (GenBank: GAQ34856.1, sharing 52% protein sequence identity with AGDC1) diffracted to 1.8 Å resolution, but had 18 protein molecules in AU, in the space group C2. Finally, the AGDC1 homolog from *Madurella mycetomatis*, PPP2 (GenBank: KXX81388.1, sharing 52% protein sequence identity with AGDC1), crystallized with only one molecule per AU in the space group P321, and its crystal structure was phased by single-wavelength anomalous dispersion (SAD) of the Se-Met derivatized protein.

Purified AGDC1 crystallized without any added ligands (apo-enzyme), except potassium from the crystallization solution, or with the substrate analog 4-nitrocatechol (4NC) in the presence of cobalt (4NC/Co^2+^ complex). Apo-AGDC1 crystallized with nine molecules in the AU, in the space group R3, forming a trimer of trimers. Apo-PPP2 had one molecule in the asymmetric unit (AU) in the space group P321 and was solved at 1.7 Å resolution by SAD (Table 1). PPP2 also co-crystallized with 4NC, and its complex structure solved at 1.6 Å resolution, was in the same space group as the apo enzyme. AGDC1 4NC/Co^2+^-complex had only one molecule in the AU in the space group P321, like the PPP2 crystal structures. The non-crystallographic symmetry present in the crystals containing multiple copies of AGDC1 in the AU resembled the threefold crystallographic symmetry in the higher symmetry space group. AGDC1 crystal structures were solved by molecular replacement, using the PPP2 4NC complex structure as a search probe. They were refined to 1.9 and 1.5 Å resolution for the apo-enzyme and 4NC/Co^2+^ complex structures, respectively (Table 1). A higher oligomeric state of AGDC1 and PPP2 was also observed in solution by size exclusion chromatography (SEC; Supplementary Fig. S4), unlike the previously reported monomer in the case of AGDC1^16^.

**Table 1:** Statistics of crystal data collection and refinement for native (AGDC1 and PPP2) and selenium-methionine derivatized proteins (PPP2-Se).

Data	AGDC1	AGDC1-APO	PPP2	PPP2-Se
PDB ID	6W54	7KD9	7JMV	7JMR
Resolution range (Å)	50–1.50	50–1.94	50–1.57	50–1.67
Space group	R 3	P 21 21 2	P 3 2 1	P 3 2 1
Unit cell (Å)	81.97, 81.97, 103.1	91.63, 265.3, 93.73	98.74, 98.74, 62.29	98.85, 98.85, 62.38
Unit cell (deg)	90, 90, 120	90, 90, 90	90, 90, 120	90, 90, 120
Wavelength (Å)	1.23984	1.0	0.97934	0.97934
Beam lines	GM/CA-CAT, APS	GM/CA-CAT, APS	GM/CA-CAT, APS	GM/CA-CAT, APS
Number of measurements	148,680	827,460	481,052	675,504
Number of unique reflections	36,568	168,891	49,087	40,823
Completeness of data (%)				
Overall	88.5	99.5	99.9	99.6
Shell/resolution range (Å)	40.2 (1.53–1.50)	96.8 (2.00–1.94)	99.5 (1.60–1.57)	98.8 (1.73–1.67)
	99.2 (50–1.69)			
R_sym_ (%)				
Overall	9.4	9.0	8.0	7.0
Last shell/resolution range (Å)	35.7 (1.53–1.50)	90.2 (2.00–1.94)	89.4 (1.60–1.57)	70.3 (1.73–1.67)
CC1/2				
Last shell/resolution range (Å)	0.819 (1.53–1.50)	0.695 (2.00–1.94)	0.795 (1.60–1.57)	0.762 (1.73–1.67)
I/sigma				
Overall	42.9	15.2	41.4	38.9
Last shell/resolution range (Å)	5.8 (1.53–1.50)	1.4 (2.00–1.94)	1.8 (1.60–1.57)	1.6 (1.73–1.67)
Wilson B-factor	18.29	33.46	20.35	27.8
*SAD—figure of merit (%)*				52.0
R_work_	14.03	19.10	18.17	17.77
R_free_	16.62	23.75	19.34	20.12
Number of atoms	2057	17,583	1942	1979
Macromolecules	1845	16,819	1805	1813
Ligands	13	3	14	4
Solvent	199	761	123	162
Mean B (Å^2^)	25.0	50.28	24.1	32.8
RMS (bonds)	0.008	0.007	0.010	0.010
RMS (angles)	0.97	0.91	1.04	1.06
Ramachandran favored (%)	97.72	95.72	97.26	96.82
Ramachandran allowed (%)	2.28	4.23	2.74	3.18
Ramachandran outliers (%)	0	0.05	0	0
Rotamer outliers (%)	0	0	0	0

*R*_*sym*_ = Σ_j_ Σ_i_ |*I*_*ij*_—< *I*_*j*_ >| / Σ_i_ Σ_j_ *I*_*ij*_, where i runs over multiple obervations of the same intensity, and j runs over all crystallographic unique intensities. R_factor_ = Σ ||*F*_*obs*_|—|*F*_*calc*_|| /Σ |*F*_*obs*_|. R_free_ was calculated with 5% of the reflections selected.

### Overall structures of AGDC1 and PPP2

**Figure 3:**
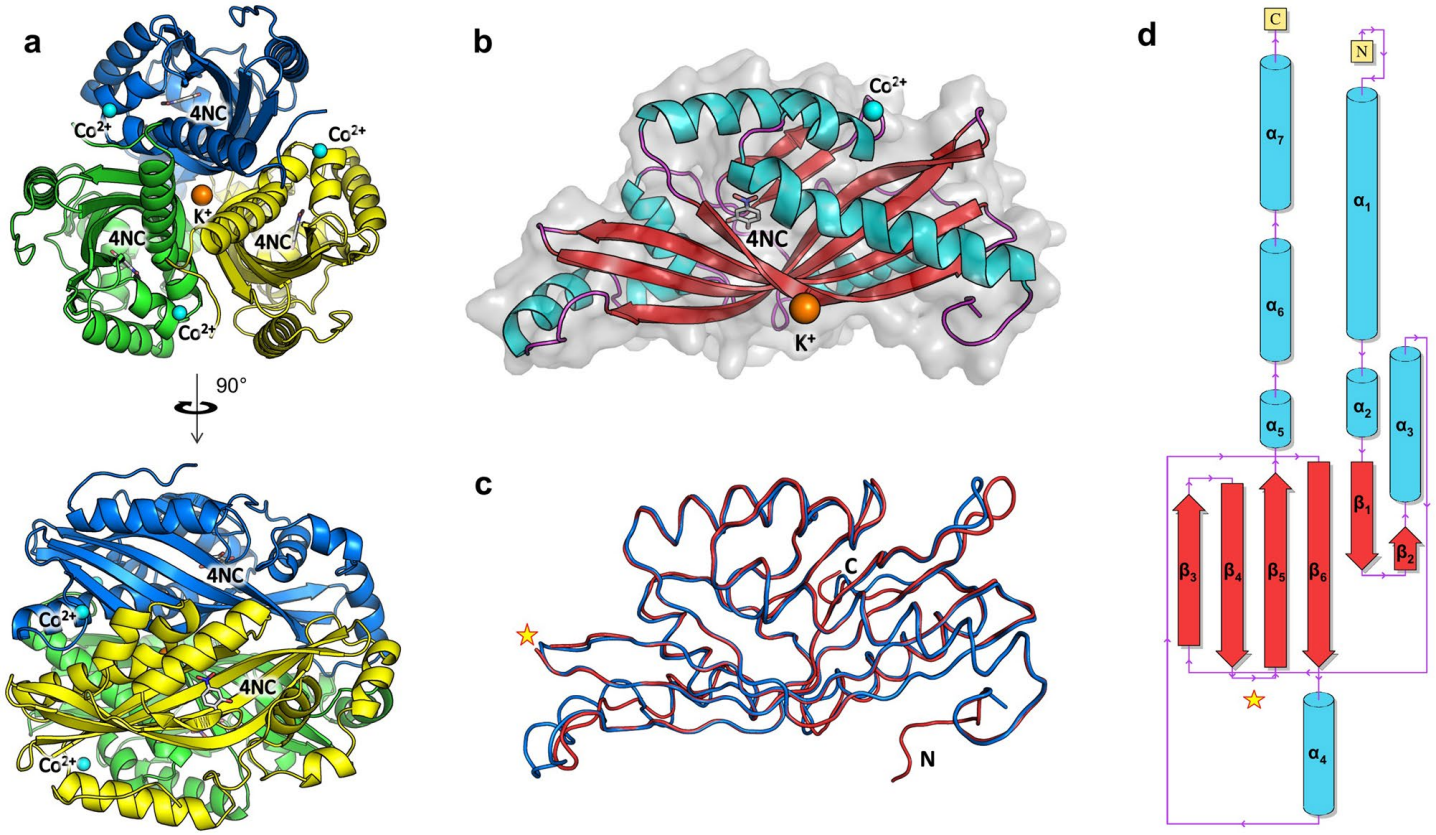
The crystal structures of AGDC1 and PPP2. (**a**) The crystal structure of the 4NC/Co^2+^ complex of AGDC1 reveals a trimeric organization of the enzyme with a central potassium ion (orange sphere) and three peripheral cobalt binding sites (cyan spheres). The substrate analog 4-nitrocatechol (4NC) binds in a deep cleft, partially delimited by the β-barrel core. Each monomer is colored differently (blue, green, or yellow). (**b**) The monomer has an elongated shape, featuring an α + β fold, with a central β-barrel and peripheral helices. α-helices, β-strands, connecting loops, and the three-dimensional space-filling model are colored in cyan, red, purple, and light grey, respectively. 4NC is in ball-and-stick representation. (**c**) Superposition of the liganded structures of AGDC1 (blue) and PPP2 (red), in the same view as in (**b**). ‘N’ and ‘C’ indicate the N- and C-termini. The star indicates the equivalent position of the additional flexible loop in PPP2, with missing electron density. (**d**) Secondary structure topology diagram of AGDC1. The color scheme is as in (**b**).

In all crystal structures, the center of the AGDC1 trimer contains a potassium ion. Besides the interaction through the potassium ion, the protomers interact via an extended interface of 10,300 Å^2^ buried surface area (Fig. 3a). The monomer displays an α + β fold, comprising a six-stranded, antiparallel β-barrel core associated with seven α-helices (Fig. 3b,d). The substrate binds in a deep cleft within the β-barrel. In the 4NC/Co^2+^ complex, the cobalt binding sites link the N-terminal regions of adjacent protomers.

PPP2 has high protein sequence homology to AGDC1, with 63% overall similarity and 51% identity (Supplementary Fig. S5). Expectedly, the PPP2 crystal structure matches closely that of AGDC1 (C_α_ RMSD: 1 Å, Fig. 3c). The main difference arises from an additional flexible, 17 residue-loop between β-strands β4 and β5 of PPP2, which had no associated electron density in the crystal structure. In contrast, helix α4 of AGDC1 is absent in PPP2, being replaced instead by a shorter segment without a secondary structure. While the potassium ion is also at the center of the PPP2 trimer, no divalent metal-binding sites, equivalent to those of Co^2+^ in AGDC1, were found in the PPP2 structure, conforming with the enzyme activity being unaffected by Co^2+^.

### The substrate-binding site

**Figure 4:**
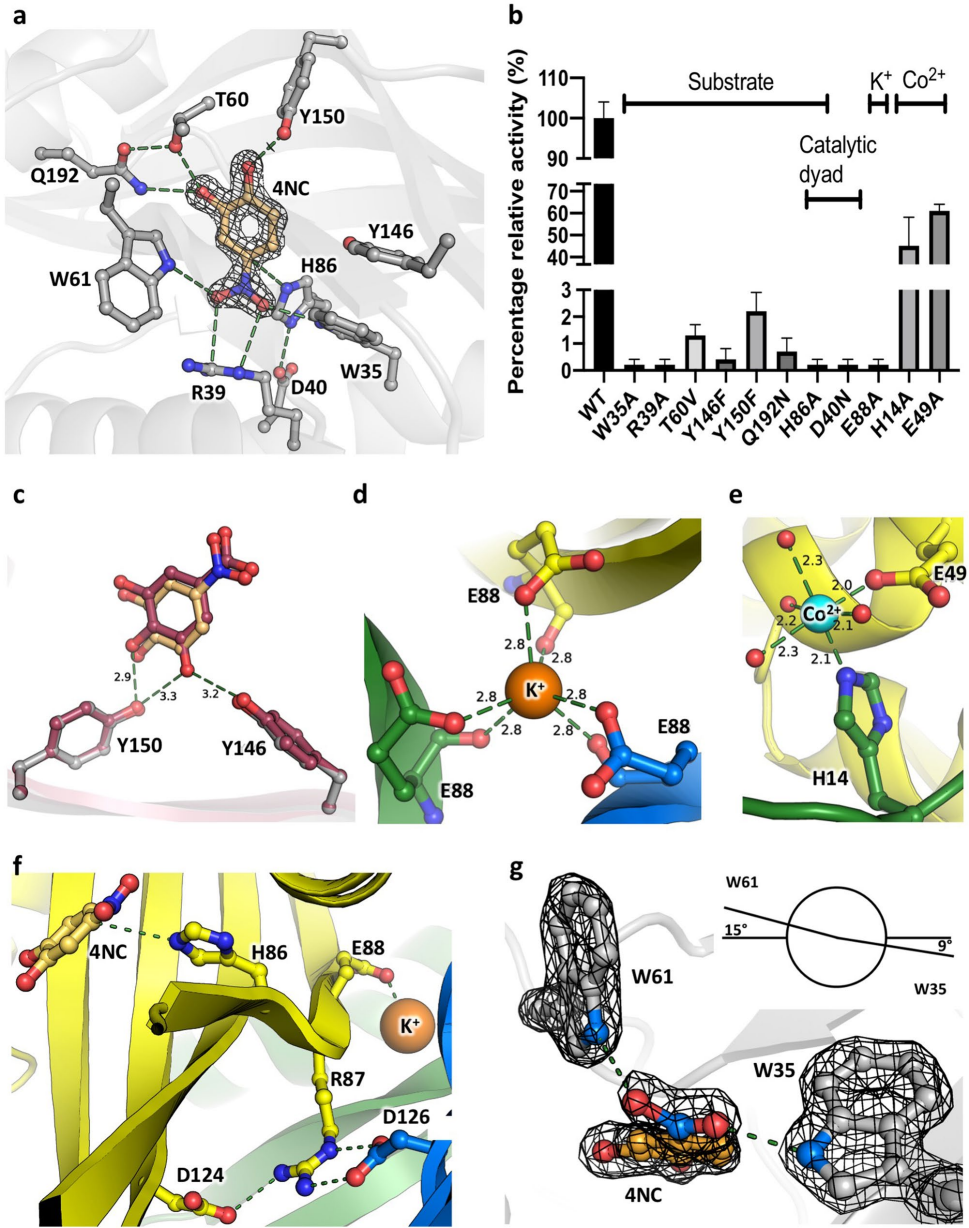
The ligand-binding sites in AGDC1. (**a**) The active site of AGDC1 with bound 4-NC. The 2Fo-Fc density map for 4NC is shown as an isosurface mesh contoured at 2σ. (**b**) Percentage enzyme activity of mutants relative to that of the wild-type. The assay had 0.5 mM gallate, 50 mM KPi, pH 6.5, 250 µM CoCl_2,_ and 40 µg/ml protein. The labels on top indicate the role of mutated residues—ligand binding or catalytic mechanism. (**c**) Induced-fit docking of the natural substrate gallic acid (GA). The crystal structure (colored grey for protein residues and gold for 4NC) is overlaid with the docking results (colored red for the protein residues and GA). K^+^ (**d**) and Co^2+^ (**e**) binding sites. (**f**) The substrate- and K^+^-binding sites are connected through a twisted beta-strand β3, with H86 and E88 on each side of the twist. (**g**) The tryptophan clamp made up of W35 and W61 twists the nitro-moiety of 4NC with respect to the benzyl ring through hydrogen bond interactions between the amines of the respective indole rings and the oxygen atoms of the ligand nitro-group. A twist of up to 24° (PPP2) could be observed in the crystal structures directly. W35, W61, and 4NC are shown in 2Fo-Fc electron density contoured at 2σ. The Newman projection indicates the dihedral angles measured respective to the aromatic plane. Panel (g) depicts the PPP2 complex, all other panels show AGDC1. The ions are displayed as spheres (K^+^: orange, Co^2+^: cyan). Bonding interactions are indicated by green dashed lines. Distance labels are in Å.

The substrate-binding site is a closed cavity within the β-barrel (Fig. 3). All atoms of the substrate analog 4-nitrocatechol (4NC) are visible in the electron density (Fig. 4a). The binding pocket is mainly hydrophobic but contains several key hydrophilic residues. R39 anchors 4NC in the active site through an interaction with the negatively charged nitro-group, and the hydroxyl groups of 4NC form hydrogen bonds with Q192, T60, and Y150. H86 points to the *si*-face of the ligand and interacts with D40, forming a catalytic dyad. The nitro-group of 4NC is rotated by ~ 24° with respect to the catechol ring through interactions of the oxygen atoms with the nitrogen-hydrogens of two conserved tryptophan residues, W35 and W61, which create a tryptophan-clamp (Fig. 4a,g). Mutations of W35, R39, T60, H86, and Q192 in AGDC1 knock-out the enzyme activity (Fig. 4b), consistent with these residues’ role in recognizing the substrate.

Surprisingly, the active site in the apo-structure has the same geometry as the 4NC complex structures. Here, however, several ordered water molecules occupy the space of 4NC. Although the exact geometry varies between the nine protein chains present in the asymmetric unit, active site residues R39, H86, W61, T60, Y150, Y146, and Q192 are involved in hydrogen-bond interactions with the ordered water molecules (Supplementary Fig. S6).

To investigate the interactions between AGDC1 and its native substrate, we docked gallic acid into the active site of the 4NC/Co^2+^-complex structure using an induced fit procedure. Here, the gallate’s binding mode was virtually identical to that of 4NC (Fig. 4c). However, the additional hydroxyl-group present in gallate forms hydrogen-bonds with two more residues: Y146 and Y150, while Y146 does not interact with 4NC in the crystal structure.

The active site residues of PPP2 and AGDC1 are very similar, with an all-atom RMSD of 0.24 Å. However, PPP2 lacks a hydrogen-bond donor equivalent to Y146, which interacts with one of the substrate’s hydroxyl groups according to the docking experiment (Fig. 4c). In PPP2, this position is a valine, with an accompanying mutation of V148 (AGDC1) to L165 (PPP2), possibly to preserve the overall cavity volume. Gallic acid is a poor substrate in PPP2 compared with AGDC1 (Fig. 2c,d). In turn, PPP2 is reported to be more active than AGDC1 on proto-catechuic acid *in vivo*^17^ PCA differs from gallic acid by the hydroxyl group interacting with Y146 (Figs. 1, 4c).

Given the minimal differences in the active site between the apo-enzyme and 4NC complex structures, we checked the substrate’s possible access pathways to the active site. The substrate-binding site appears to be a sealed cavity in the central β-barrel (Fig. 3a). Analysis of the AGDC1 structure with the program CAVER^29^ revealed two possible substrate entry pathways, passing through a second, upstream cavity (Supplementary Fig. S7). The two pockets are connected by a very narrow bottleneck, with a radius of 0.8 Å, mainly formed by I84, a residue conserved in both AGDC1 and PPP2. In silico analysis of different rotamers together with in silico glycine and alanine mutations readily opened the substrate channel. So, I84 might block substrate access to the binding site in the crystal structure, but even subtle protein-breathing should be sufficient to open the channel in solution.

### The potassium binding site

All structures contain a central metal-binding site, formed by three equivalent glutamate residues (E88), one from each monomer, which coordinate the metal with side chain and carbonyl oxygen interactions in a distorted octahedron (Fig. 4d). The distances and geometry are consistent with the metal being a potassium ion, as confirmed by the Metal Binding Site Validation Server^30^. The potassium source is probably the high concentration (200 mM) of potassium salts (potassium thiocyanate or potassium formate) present in the crystallization solutions. AGDC1 also crystallized with sodium malonate and PEG 3350 (without potassium), but it diffracted only to 5 Å. The potassium coordination by E88 is associated with a local twist in the outmost beta-strand (β3), which orients the crucial histidine H86 towards the active site (Fig. 4f). The twist is further stabilized by salt bridge interactions of R87 with D124 from the same monomer and D126 from another monomer. The potassium coordination is essential for activity, as indicated by the requirement of having KPi present in the activity assay (Fig. 2a) and the lack of enzyme activity in the E88A mutant (Fig. 4b). Despite being at the trimerization interface and crucial for activity, potassium does not seem to be essential for oligomerization, as evidenced by the extensive intermonomer interactions and the size exclusion chromatography (SEC), which showed trimers for the E88A mutant and the wild-type fractionated in buffer lacking potassium (Supplementary Fig. S4). E88 is conserved in PPP2 (Supplementary Fig. S5), and the potassium binding site in PPP2 is identical to that described for AGDC1, but the occupancy of potassium in PPP2 was 19% compared to 100% for AGDC1 after occupancy refinement in Phenix^31^. The lower potassium occupancy in PPP2 is probably due to the differences in crystallization conditions: 200 mM potassium salt for AGDC1 vs. its absence (200 mM CaCl_2_ instead) for PPP2 crystals.

### The cobalt binding site

The cobalt binding site in the crystal structure was identified by anomalous X-ray scattering. The cobalt ion is octahedrally coordinated by E49 and H14, each from a different monomer, located at the N-termini of neighboring chains, and four water molecules. The coordination bond lengths and geometry are consistent with the metal being cobalt^30^. In the apo-structure, which lacked cobalt in the crystallization buffer, H14 and E49 interact directly via a hydrogen bond. The cobalt binding site of AGDC1 is missing in PPP2, in which a serine side chain (S52 in PPP2) replaces the crucial E49 (Supplementary Fig. S9). Cobalt increases AGDC1 activity up to threefold (Fig. 2b, Supplementary Fig. S1b), and single mutations in the cobalt binding site reduce the activity by 40–60% (Fig. 4b), indicating a role of cobalt binding on enzyme activation. Furthermore, the optimum pH of the cobalt activation is consistent with the pK_a_ of His, further underlining the importance of H14 for cobalt binding (Supplementary Fig. S3).

To our knowledge, no similar binding sites for divalent metals were reported for this enzyme class, except for the structure of a putative scytalone dehydratase from *Novosphingobium aromaticivorans* (PDB ID 3ef8), which binds magnesium through a similar N-terminal aspartate. However, this interaction seems to facilitate crystal contacts rather than contribute to trimerization.

### The proposed reaction mechanism for AGDC1 and PPP2

Inspecting the AGCD1 active site showed that H86 is in a favorable orientation for catalysis, with its τ-nitrogen points towards the C4 carbon of the substrate (Fig. 4a). A search for structurally homologous proteins in the PDB (http://www.rcsb.org) with the DALI server^32^ revealed that the H86-D40 dyad is conserved in at least three characterized lyases: gamma-hexachlorocyclohexane dehydrochlorinase LinA^33^ (PDB ID 3A76), scytalone dehydratase^34^ (PDB ID 4STD-7STD), and bile-acid 7-alpha dehydratase (PDB ID 4LEH). In the structures of the LinA and scytalone dehydratase, the dyad acts via an acid–base mechanism.

**Figure 5:**
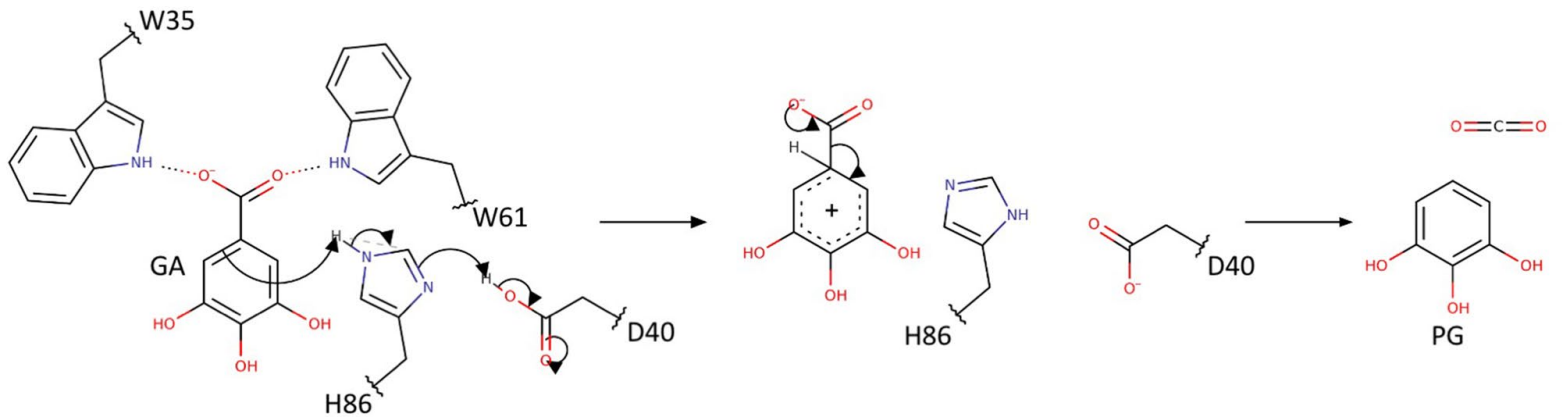
The proposed catalytic mechanism for AGDC1 and PPP2. The tryptophan-clamp (W35 and W61) leads to a rotation of the substrate carboxyl group, thus increasing the energy of the substrate. The reaction proceeds via an electrophilic aromatic substitution (S_E_Ar). H86, activated by D40, protonates gallate to form a Wheland intermediate. Elimination of CO_2_ completes the reaction by producing pyrogallol. The reaction from protocatechuic acid to catechol proceeds the same way.

The here proposed catalytic mechanism for the non-oxidative decarboxylation of gallate forms a hybrid between acid–base catalysis and transition state stabilization (Fig. 5). Judging from the geometry of the binding pocket (Fig. 4a,g), the clamping of the carboxyl-group by the tryptophan residues W35 and W61 could lead to a ~ 30º rotation of the carboxyl-group around the C–C bond. A slight out-of-plane rotation by 7 and 24° also emerged during the automatic stages of structure-refinement for AGDC1 and PPP2, respectively, for the nitro group of the co-crystallized substrate analog 4NC. The rotation can occur because the benzene ring is immobilized. Its hydroxyl groups are held in a tight network of hydrogen bonds with the T60/Y146/Y150/Q192 residue cluster at the opposite side of the molecule (Fig. 4a,c). The carboxyl group’s out-of-plane rotation relative to the restrained benzene ring delivers the energy to overcome the reaction barrier by “shearing” the substrate. Density functional theory (DFT) calculations on gallic acid support these considerations. They reveal that a tilted conformation of the carboxyl-group is destabilizing the molecule due to the disruption of π-π interactions between the C-O double bonds and the aromatic plane^7,35^. This conformation resembles radical species^35^. Calculations on silver and copper-catalyzed decarboxylation of benzoic acids also showed the deformation of the planar geometry to be crucial for the reaction^36^. The reaction, now with the substrate prone for decarboxylation, likely proceeds via an electrophilic aromatic substitution (S_E_Ar). H86, activated by the hydrogen bond with D40, protonates gallate at the C4 position to form the Wheland intermediate. Aromaticity can be reestablished by the elimination of CO_2_, thus producing pyrogallol.

The exact order of events remains hypothetical and has to be investigated by a closer kinetic/spectroscopic investigation. All the residues mentioned to be critical for catalysis are also present in PPP2, establishing the commonality in the reaction mechanism for the two homologs. Mutants affecting the catalytic dyad (H86A, D40N) or carboxyl group orientation (W35A) abolish enzyme activity, supporting the proposed critical roles of these residues in the reaction mechanism (Fig. 4b). Additionally, the pH optimum for enzyme activity (6.5–7.0) is close to the pK_a_ of His in a catalytic dyad with aspartate (~ 6.2^37,38^), in line with the role proposed for H86.

## Discussion

Polyphenolic acid decarboxylation is a much-desired reaction in biotechnology. AGDC1 and PPP2 facilitate this reaction in different fungal species. Intriguingly, these enzymes’ catalytic mechanism is a new type of decarboxylation mechanism combining acid–base catalysis by a His-Asp dyad with a sophisticated web of hydrogen bonds and a double tryptophan twisting clamp.

The trimers of AGDC1 and PPP2 in the crystal structures represent the functional biological unit, evidenced by the size exclusion chromatography data and the extensive intermonomer buried surfaces and interactions. The trimer seems to be stabilized by the metals: the central potassium ion bridges the monomers through interactions with E88 from each protomer. In AGDC1, cobalt additionally seals the N-termini of adjacent monomers by bringing in its coordination sphere H14 from one monomer and E49 from another. The enzyme assay indicates that potassium is essential for AGDC1 activity, and cobalt further activates the enzyme. A large group of non-oxidative decarboxylases uses metals directly for catalysis^19^, whereas AGDC1 uses potassium and cobalt indirectly to pre-organize the active site.

The activation by cobalt appears to occur through an allosteric mechanism since the active site and the cobalt-binding site are far removed (Fig. 3a). The structures with and without bound cobalt show identical conformations, but mutating the cobalt binding residues reduces enzyme activity in the cobalt-containing buffer. The protein coordinates cobalt by occupying only two of the six coordination sites, indicating a rather weak interaction. The pH dependence of cobalt-activation follows the pH optimum of AGDC1 cooperatively, with the highest activation observed at the pH optimum but almost no activation in the upper and lower pH ranges. These two effects are consistent, as both cobalt binding and catalysis are facilitated by histidine residues (H14 and H86, respectively), with an expected pKa of ~ 6.8 for surface-exposed histidine residues^38^. A mechanism for allosteric crosstalk between the N-terminal cobalt binding site and the active site is unclear from the crystal structure. Notably, cobalt is a rare metal in yeast, where it fails to reach µM levels^39^. Perhaps, the native ligand is a divalent metal with a higher abundance, like zinc^39^. However, zinc partially inhibited the enzyme activity, and other divalent metals such as MgCl_2_, MnCl_2_, or CaCl_2_, also failed to activate or even inhibited the enzyme (Supplementary Fig. S1). Therefore, both the role and mechanism of cobalt activation remain obscure and could be irrelevant in vivo. However, knowledge about cobalt activation could be valuable for future enzyme-engineering approaches.

On the other hand, the structural data hint towards a potential role for the potassium-binding site in the regulation of enzyme activity, as the β3-strand shows a twisted conformation localized close to E88, which coordinates the metal not only through the side-chain but also through the backbone carbonyl interactions (Fig. 4d). The right-handed twist seems to be induced by the potassium binding (Fig. 4f), bending the β-sheet by 180º and flipping the subsequent residues to the opposite facet of the strand. Twisting of β-sheets is a commonly found motif in globular proteins, and its origin is an active field of research but still not completely understood^40,41^. The reported natural tendency of β-strands to form right-handed twists is rather subtle and usually does not exceed 30º per residue^42^. In contrast, the here observed potassium-facilitated twist is ~ 45º per residue. To our knowledge, this is the first structure in which ion coordination seems to play a direct functional role in beta-strand twisting. Potassium-binding induced beta-strand twist exposes R87 (the residue next to E88) to the inter-chain contact surface, locking the β3-strand twisting conformation through R87 salt bridges with D124 of the same monomer and D126 of the adjacent protomer. The next residue is H86, which is essential for the reaction catalysis (Figs. 4, 5). Through the β3-twisted conformation, H86 faces the inside of the β-barrel, pointing into the active site. So, potassium binding is priming the active site for catalysis. This observation can explain why potassium is essential for activity (E88A is a dead mutant), but not for trimerization, as both wild-type and E88A proteins run as a trimer on size exclusion in buffers with and without potassium (Supplementary Fig. S4).

Interestingly, the related structure of LinA , which is also a trimer, does not contain the central metal-binding site. Its equivalent β-strand lacks the twist, further implying that potassium is not required for oligomerization but in organizing the active site in AGDC1 and PPP2. The same holds for the scytalone dehydratase, whose fold resembles that of AGDC1 and PPP2 even closer (PDB ID 3EF8) (Supplementary Fig. S8). The organizational role of potassium may also explain the high similarity in the active sites of the apo-enzyme and the 4NC complex structures, both liganded with potassium present in crystallization conditions. Finally, the very similar organization of the active site of AGDC1/PPP2, LinA, and scytalone dehydratase implies that this type of enzyme can support a large variety of substrates.

LinA displays the same core-fold as AGDC1/PPP2 but lacks extended C- and N-terminal sections (Supplementary Fig. S8). The high divergence on the sequence level (sequence identities of LinA and scytalone dehydratase to AGDC1 and PPP2 vary between 4–14%) likely prevented us from finding the structures when we initially searched for MR phasing probes. So, the conserved core-fold seems to be very tolerant for large additions and high sequence divergence. Together with its substrate promiscuity, the fold may serve as a promising template for designer-enzymes.

AGDC1 and PPP2 have very similar structures, with some key exceptions. First, PPP2 lacks the cobalt binding site since D49 of AGDC1 is S52 in PPP2 (Supplementary Fig. S9), consistent with the insensitivity of PPP2 activity to Co^2+^ (Fig. 2e); second, it has a long and flexible loop between the β-strands β4 and β5; and third, it lacks a potential hydrogen-bond interaction with one of the hydroxyl groups of gallate, as Y146 of AGDC1 is V165 of PPP2 (Fig. 4c). While this active-site substitution explains why AGDC1 shows a higher activity towards gallic acid (GA) than PPP2, it is irrelevant for the alternate substrate protocatechuic acid (PCA), which lacks this very hydroxyl group. Indeed, PPP2 is reported to be more active with PCA than AGDC1 *in vivo*^17^, and in AGDC1, the K_M_ for GA is lower than for PCA^16^, a difference possibly stemming from the additional interaction that GA has with Y146, compared with PCA.

Considering the structural differences between PPP2 and AGDC1, a possible explanation for the higher activity of PPP2 on PCA compared with AGDC1 without Co^2+^ is that increased flexibility in the linking regions of the beta-strand core that houses the active site may lead to higher activity. Another possibility is that PCA may bind in two different ways in AGDC1. If PCA binds with its hydrophilic meta hydroxyl group facing the hydrophobic valine residue (V165), it will disrupt the substrate’s tight coordination. The benzene ring will not be constrained enough for the tryptophan-clamp to apply a shear to the substrate. In contrast, a binding mode flipped by 180° would not suffer from this unfavorable interaction. Here, the meta hydroxyl group would be locked by hydrogen bonding with Q192. The possibility of two dual mutually exclusive binding modes creates an unusual situation where one binding mode of PCA would make the substrate become its competitive inhibitor at the same time. Multiple binding modes of one substrate in an enzyme’s active site are not unpreceded^43^. No such issue arises for GA, in which both orientations are indistinguishable.

To our knowledge, the reaction mechanism proposed here is a new mode of enzymatic decarboxylation of polyphenolic compounds. An interplay of an aspartate-histidine dyad and substrate destabilization facilitates the reaction. On the one hand, the potassium ion bound to the trimer core twists β-strand β3, orienting the catalytic dyad towards the active site. On the other hand, a pair of tryptophans (W35 and W61) destabilizes the substrate by twisting the carboxyl group, while a network of hydrogen bonds hold the phenol ring in place at the same time. Aspartate-histidine dyads are a common motif in enzymology and are also part of similar structures, like LinA and the scytalone dehydratase. However, the structures are still mechanistically different. AGDC1′s dyad is an acidic, ribonuclease A-type^44^ dyad, where aspartate protonates the crucial histidine residue. In contrast, aspartate deprotonates histidine in LinA and scytalone dehydratase, to increase its basicity.

The results obtained by the activity assay confirm the proposed role of H86 as a key-catalytic residue, with the H86A mutant completely losing its activity. The inactivation of AGDC1 upon mutation of W35 is also consistent with the proposed reaction mechanism, as W35 plays a crucial role in activating the substrate. However, the tryptophan to alanine mutation supposedly has a significant impact on the overall binding pocket geometry, which could also account for the observed effect. Finally, the activity-loss upon mutating R39 can be explained by a diminished substrate affinity; the ionic interaction is likely the driving force of substrate binding.

The original publication describing AGDC1 identified several homologs in lactic bacteria^16^. However, the side chains relevant for catalysis, especially the crucial histidine residue, seem to be absent in the presented alignment. We conclude that these homologs likely do not resemble the same type of gallic acid decarboxylases.

In summary, this study provides the first insight into the structure–function relationship of a recently discovered class of non-oxidative decarboxylases. Our work establishes the structural underpinnings of a new reaction mechanism for cofactor-independent decarboxylases, amenable to various biotechnological applications. It also provides a molecular basis for engineering these enzymes to recognize other substrates, increase specificity where side-reactions are undesirable and speed up their reaction.

## Materials and methods

### Protein expression and purification

AGDC1 (GenBank: SJN60119.1) and PPP2 (GenBank: KXX81388.1) DNAs were synthesized by GenScript Biotech (Piscataway, NJ, USA). The genes were cloned in pET15b (Merck KGaA). Proteins were expressed in *E. coli* C41 cells^45^, cultured in Luria Bertani media containing 50 µg/ml ampicillin, at 37 °C, with shaking (200 rpm). For selenomethionine labeled protein expression, cells were grown in M9 media with amino acid supplements as previously described^46^. Protein expression was induced by the addition of isopropyl β-D-1-thiogalactopyranoside at a final concentration of 0.4 mM when cell solution OD_600nm_ reached 0.9. Three hours after induction, cells were collected by centrifugation (4,000 × g, 5 min). The cell pellet was resuspended in the lysis buffer (50 mM Sodium phosphate, pH 7.5, 200 mM NaCl, 5% (v/v) glycerol) containing ~ 0.25 mg/ml lysozyme and frozen at -20 °C. After thawing, the cell solution was supplemented with deoxyribonuclease I (0.1 mg/ml), MgCl_2_ (2 mM), and phenylmethylsulfonyl fluoride (2 mM), and cells were broken by sonication (Branson Ultrasonics, Danbury, CT, USA). The broken cell solution was centrifuged at 9,000 × g, for 30 min, at 4 °C. Imidazole was added to the supernatant to a final concentration of 2 mM; then, the protein solution was brought to pH 7.5 with 0.5 M NaOH before final centrifugation (120,000 × g, 30 min, 4 °C). The supernatant was loaded onto a Ni–NTA column (Qiagen, Germantown, MD, USA), pre-equilibrated with Buffer A (50 mM NaPi, pH 7.5, 500 mM NaCl, 5% (v/v) glycerol) containing 5 mM imidazole. The column washes consisted of: 100 ml Buffer A with 5 mM imidazole, 40 ml Buffer A with 10 mM imidazole, and 40 ml Buffer A with 20 mM imidazole. Protein was eluted with 50 mM NaPi, pH 7.5, 200 mM NaCl, 5% (v/v) glycerol, 300 mM imidazole. The eluted protein was 95% pure as judged by SDS-PAGE. Removal of the terminal Histidine tag was performed with thrombin (10 units/mg target protein, Biopharm Laboratories, LLC, Bluffdale, UT, USA), overnight at 4 °C, in the lysis buffer. The protein was reloaded on a Ni–NTA column. The protein without the Histidine tag (i.e., the pass-through) was concentrated with 10 K cut-off Amicon centrifugal filters (Merck KGaA) and dialyzed overnight at 4 °C in 25 mM Tris pH 7.5. Final protein solutions of at least 20 mg/ml concentration were flash-frozen in liquid nitrogen as aliquots of 50 µl and stored at -80 °C until needed.

### Protein crystallization and structure determination

AGDC1 and PPP2 crystals were obtained by hanging drop vapor diffusion. The crystallization drop contained one microliter of protein solution at 10 mg/ml in 20 mM Tris, pH 7.5, with or without ligands (5 mM nitrocatechol alone or with 0.5 mM CoCl_2_), and one microliter of precipitant solution. For AGDC1, the apo enzyme crystallized in 24% (w/v) PEG 3350, 0.2 M Potassium thiocynate, 0.1 M MES pH 6.0, and the NC/Co^2+^ complex crystallized in 28% (w/v) PEG 3350, 0.2 M Potassium formate, 0.1 M MES pH 6.5. For PPP2, the precipitant solution consisted of 15–18% (w/v) PEG 3350, 0.2 M Calcium chloride and 0.1 M MES pH 5.5. Crystals appeared at room temperature within 2–3 days. All x-ray diffraction data collection was performed at Beamline 23ID, The General Medical Sciences and Cancer Institutes Structural Biology Facility at the Advanced Photon Source, Argonne National Laboratory, Lemont, IL. Phasing for selenomethionine-substituted PPP2 crystals was obtained by single-wavelength anomalous diffraction (λ = 0.979345) with the program ShelxC/D/E^47^. Residue tracing was performed with ARP/wARP^48^ and Coot^49^. The initial model was refined with Phenix^31^ and Refmac^50^. Crystal structures of AGDC1 were solved by molecular replacement with the PPP2 model, using the program Phaser^51^. Figures were generated with PyMOL (www.pymol.org, Schrödinger, New York, NY, USA).

### Gallic acid docking

Virtual docking of gallic acid to the AGDC1 crystal structure was performed with Molecular Operating Environment (MOE, http://www.chemcomp.com, Chemical Computing Group, Montreal, QC, Canada). A gallic acid conformation library was generated with Conformation Search. AGDC1 model was prepared by protonation at pH 6.5 and energy minimization. With Dock function, gallic acid conformations were docked onto the 4NC binding site, using default parameters in Triangle Matcher, and scored by London dG and GBVI/WSA dG.

### Structural analysis

Structural figures were generated with PyMOL (www.pymol.org). Distance, angle, and buried surface were measured using PyMOL and Coot^49^. Structural alignments were conducted with PyMOL. The topology diagram was created with PDBsum^44^. Substrate channels were calculated with the CAVER PyMOL plugin^29^.

### AGDC1 and PPP2 activity assay

Enzyme activity was measured by the decrease in time of the absorbance at 259 nm for GA^16^ (Acros Organics) or at 250 nm for PCA (Beantown Chemical, NH, USA), on a Shimadzu UV–VIS spectroscope (Kyoto, Japan), at 37 °C or RT. PCA UV-spectrum scan showed two peaks: one at 287 nm and another at 250 nm (Supplementary Fig. S10). The peak at 250 nm had an absorbance more than twice that at 288 nm, therefore PCA concentration change in time was monitored at 250 nm. In 50 mM KPi, pH 6.5, 0.5 mM GA or PCA had the corresponding absorbances (i.e. Abs_259 nm_ for GA Abs_250 nm_ for PCA) at ~ 0.8 (Supplementary Fig. S10, S11). For AGDC1, after testing different conditions (various concentrations of KPi, addition of salts: CoCl_2_, CaCl_2_, MgCl_2_, MnCl_2_, ZnCl_2_, or EDTA) we found optimal enzyme activity in 50 mM KPi, pH 6.5, and 250 µM CoCl_2_. For PPP2, the assay solution was the same as for AGDC1 but without CoCl_2_.The quartz cuvette (FireflySci, Inc., NY, USA) had 0.2 cm light path and 500 µl volume, limiting the maximum substrate concentration to ~ 0.5 mM. The assay volume was 500 µl and contained 0.5 mM GA or PCA. The reaction was started by the addition of purified AGDC1 (0.04 mg/ml when the assay was at 37 °C or 0.08 mg/ml at RT) or PPP2 (0.16 mg/ml at RT). The assay time was 100 s with 10 s delay. For investigating enzyme activation by Co^2+^, we excluded 250 µM CoCl_2_ and titrated different concentrations of CoCl_2_. For 4-nitrocatechol inhibition we used 0.5 mM of the inhibitor. For activity pH optimum, the assay buffer was 50 mM KPi of pH 5.0, 6.0, 6.5, 7.0, or 8.0. Kinetic data was analyzed with GraphPad Prism (www.graphpad.com).

### Size exclusion chromatography (SEC)

To determine the molecular weight of the PPP2, AGDC1, and AGDC1 E88A, samples of each protein were analyzed by size exclusion chromatography using a Superdex 200 10/300 (Cytiva, Marlborough, MA, USA) column mounted on a Bio-rad (Hercules, CA, USA) NGC chromatography system. SEC runs of the proteins were done in the presence (25 mM Tris 7.5, 50 mM KCl, 100 mM NaCl) or absence (25 mM Tris 7.5, 100 mM NaCl) of potassium. Bio-rad Gel Filtration Standard (catalog number 1511901) was used as a standard. All proteins eluted as a single peak. Chromatograms were analyzed and plotted using a home-written Python script.

## Supplementary Information

Supplementary Information 1.
