# Supplementary material for: Crystal structures of non-oxidative decarboxylases reveal a new mechanism of action with a catalytic dyad and structural twists
Source: Sci Rep. 2021 Feb 4;11:3056. doi: 10.1038/s41598-021-82660-z (PMC7862292; doi:10.1038/s41598-021-82660-z)
Supplement: Supplementary file 1 — Supplementary Information 1. [file 41598_2021_82660_MOESM1_ESM.docx]

**SUPPLEMENTARY INFORMATION**

**Crystal Structures of Non-Oxidative Decarboxylases Reveal a New Mechanism of Action with a Catalytic Dyad and Structural Twists**

Matthias Zeug^1,3,4^, Nebojsa Markovic^2^, Cristina V. Iancu^3^, Joanna Tripp^4^,

Mislav Oreb^4,*^, Jun-yong Choe^2,3,*^

^1^Department of Chemistry, Biochemistry, and Pharmacy, Goethe University Frankfurt, Frankfurt am Main, Germany.

^2^Department of Biochemistry and Molecular Biology, The Chicago Medical School, Rosalind Franklin University of Medicine and Science, North Chicago, Illinois, USA.

^3^East Carolina Diabetes and Obesity Institute, Department of Chemistry, East Carolina University, Greenville, North Carolina, USA.

^4^Institute of Molecular Biosciences, Faculty of Biological Sciences, Goethe University Frankfurt, Frankfurt am Main, Germany.

**Corresponding Authors:** J. Choe, choej18@ecu.edu

M. Oreb, m.oreb@bio.uni-frankfurt.de

Supplementary Figure S1

Supplementary Figure S2

Supplementary Figure S3

Supplementary Figure S4

Supplementary Figure S5

Supplementary Figure S6

Supplementary Figure S7

Supplementary Figure S8

Supplementary Figure S9

Supplementary Figure S10

Supplementary Figure S11

**a b**

**Figure S1. The effect of EDTA and divalent metals on AGDC1 activity**. (a) Effect of EDTA (2 mM) and various divalent metals (250 µM) on the relative activity of AGDC1 at RT. The assay buffer had 50 mM KPi, pH 6.5, 0.5 mM GA, and 80 µg/ml AGDC1. (b) Optimal activation by 200 µM CoCl_2_, at 37 °C. The assay buffer had 50 mM KPi, pH 6.5, 0.5 mM GA, and 40 µg/ml AGDC1. Error bars represent standard deviations from at least three different measurements.

**Figure S2. Effect of temperature on the enzyme activity of AGDC1**. AGDC1 was assayed in the same conditions at 37 °C or room temperature (RT). The assay had: 50 mM KPi, pH 6.5, 0.5 mM GA, 250 µM CoCl_2_, and 80 µg/ml AGDC1. Error bars represent standard deviation and come from at least 3 different measurements.

**Figure S3. Effect of pH on AGDC1 activity.** AGDC1 (80 µg/ml) with 0.5 mM GA was assayed at RT in 50 mM KPi, at pH 5.0, 6.0, 6.5, 7.0, or 8.0, in the presence or absence of 250 µM CoCl_2_.


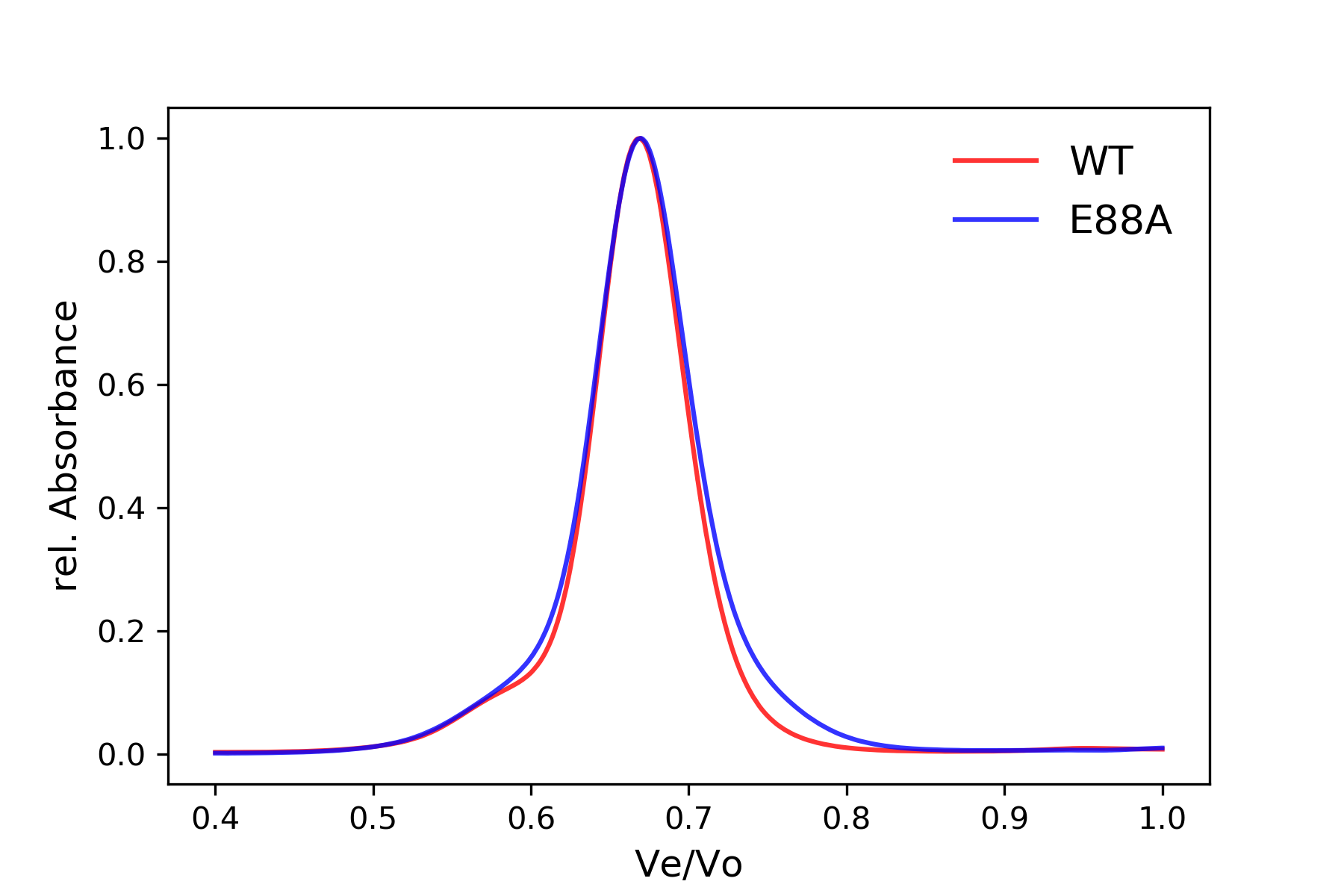

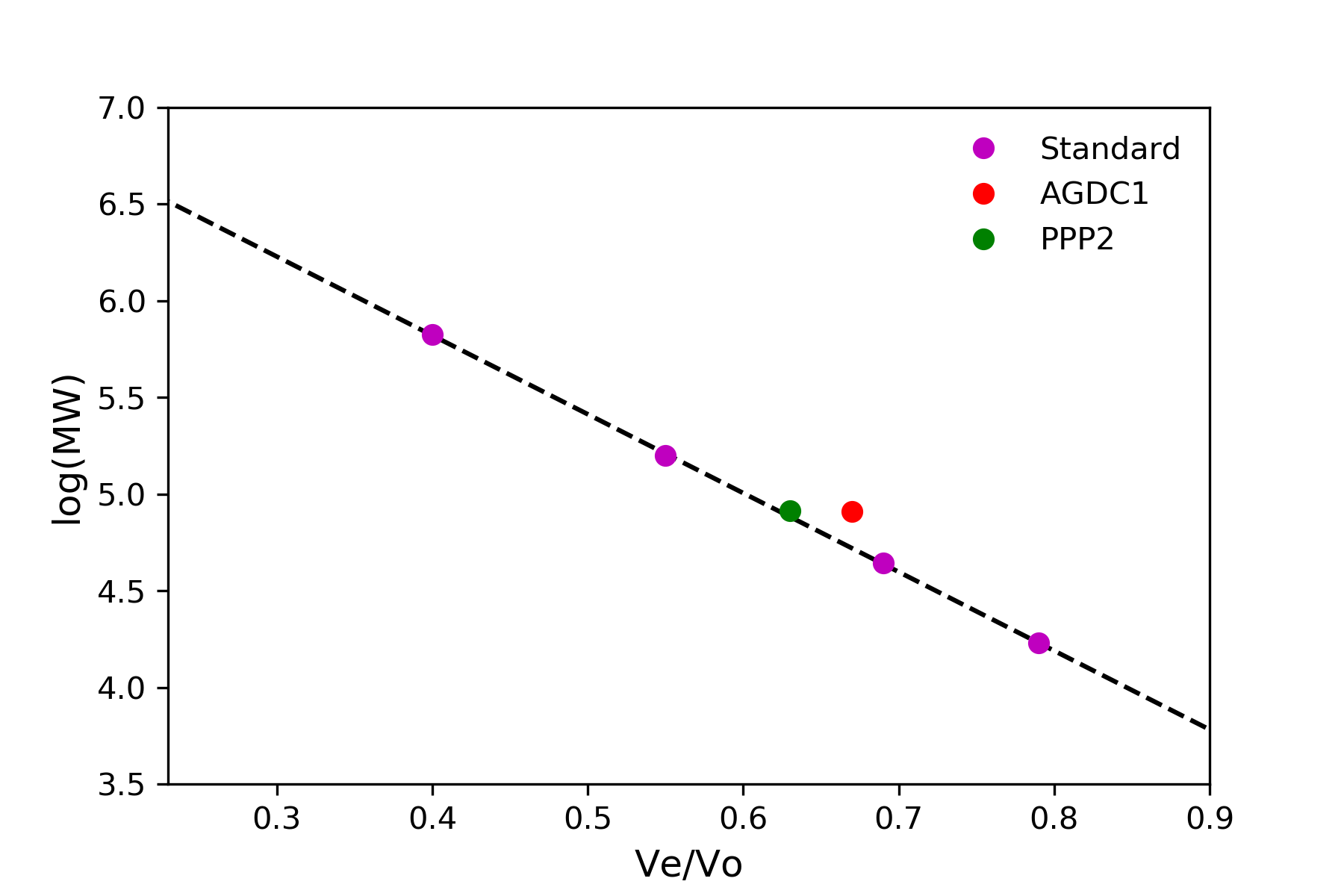


a b

**Figure S4. Size-exclusion chromatography of purified AGDC1 and PPP2.** (a) The SEC column was calibrated using the Biorad gel filtration standard. Purple dots represent the elution volume of the proteins of the standard against their respective log10 molecular weight, as given by the manufacturer’s manual. The calibration curve was derived by linear regression (R > 0.999). The molecular weight of PPP2 (green) and AGDC1 (red) was calculated according to their primary structure, showing a close agreement of calculated and measured molecular weight of PPP2 but an offset for AGDC1, indicating a slightly smaller hydrodynamic radius. (b) Chromatogram of purified wild-type (WT) and E88A AGDC1. All experiments were conducted with 25 mM TRIS pH 7.5, 100 mM NaCl as running buffer. Adding 50 mM KCl in the running buffer did not affect the chromatograms.


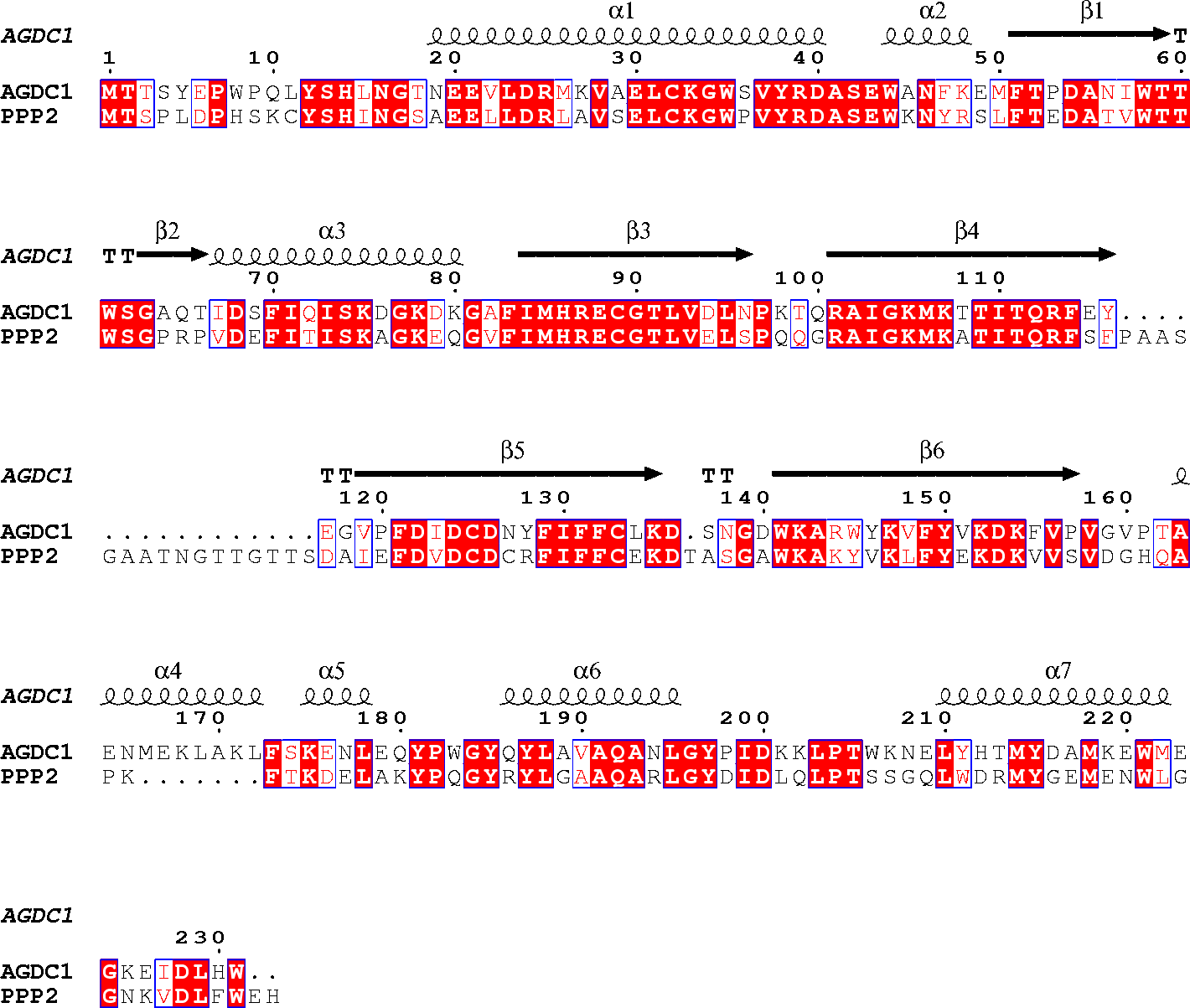


**Figure S5. Sequence alignment between AGDC1 and PPP**. The secondary structure elements (see also Fig. 3d) appear on top of the sequence.


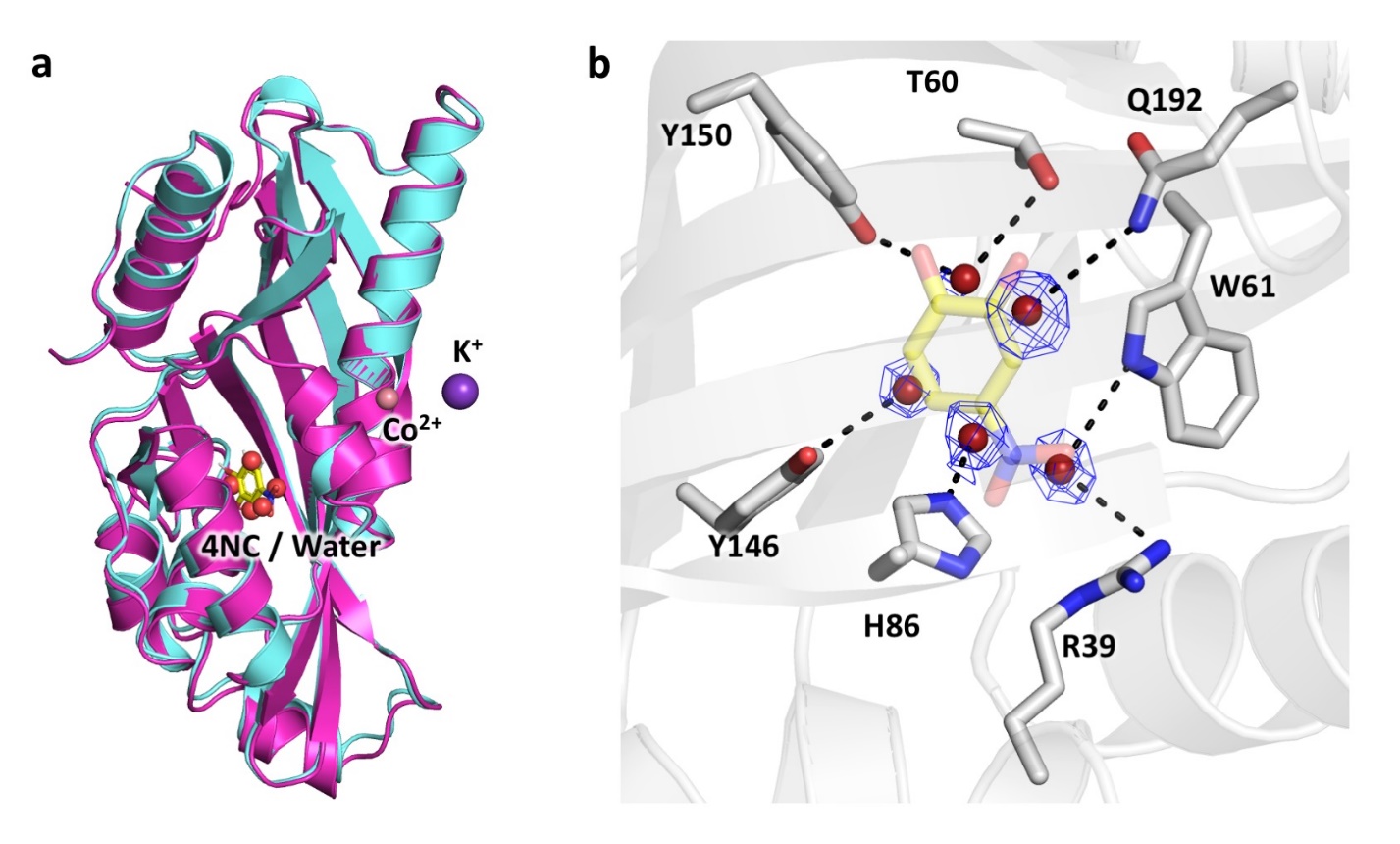


**Figure S6. AGDC1 active site in the apo crystal structure.** (a) Superposition of the apo (magenta) and liganded (cyan) AGDC1 crystal structures. 4NC (yellow) and water (red) molecules are located at the same site. (b) The 4NC site (transparent yellow) is occupied by water molecules (red) in the apo structure. 2Fo-Fc electron density map for the water molecules is shown at 1σ.


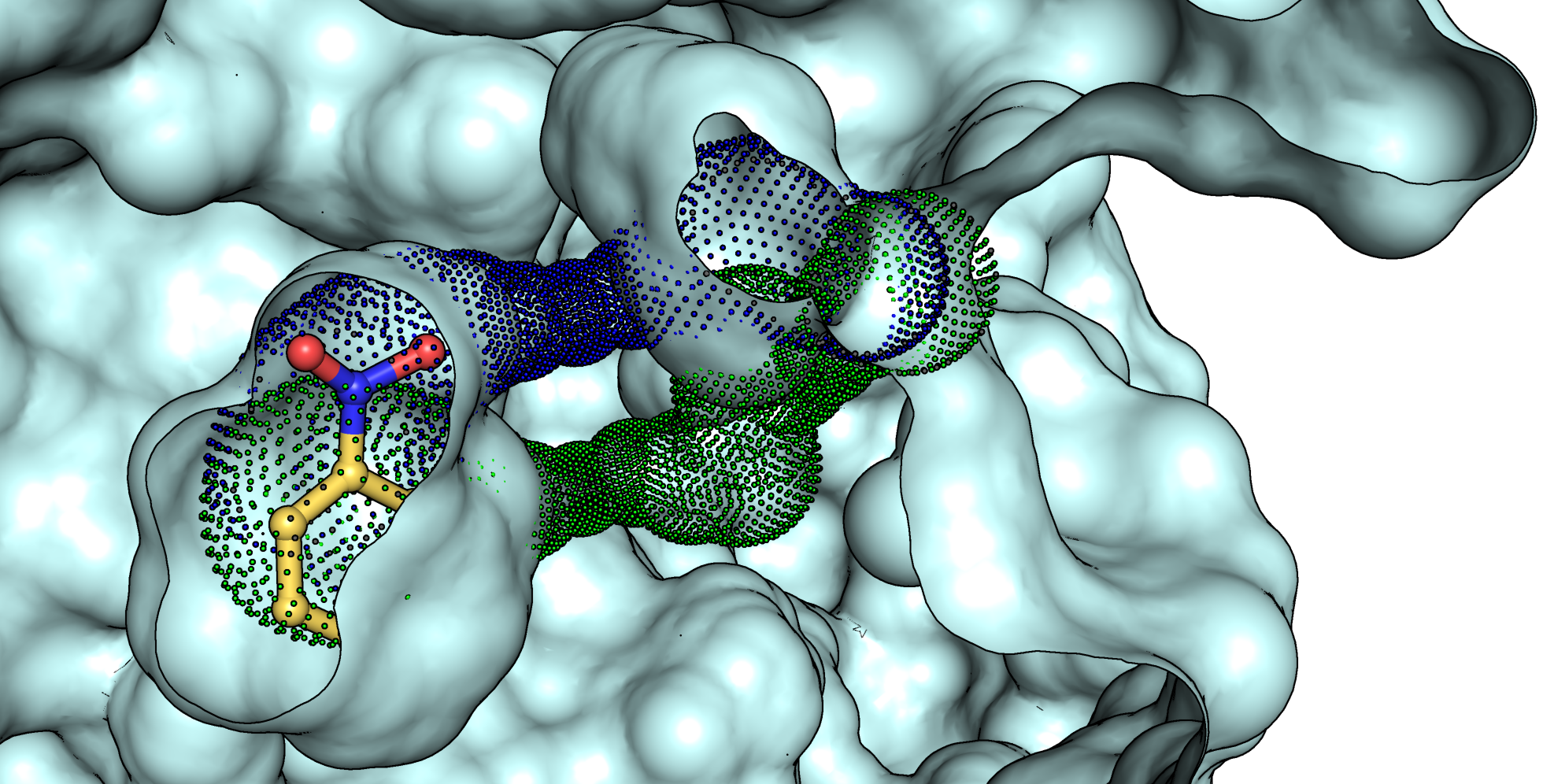


**Figure S7. Possible access pathways of the substrate to the active site.**

**Figure S7. Possible access pathways of the substrate to the active site.** Two possible access pathways of the substrate to the active site as calculated by CAVER. The red arrows indicate the central cavity of the active site (left) and the upstream cavity (right).


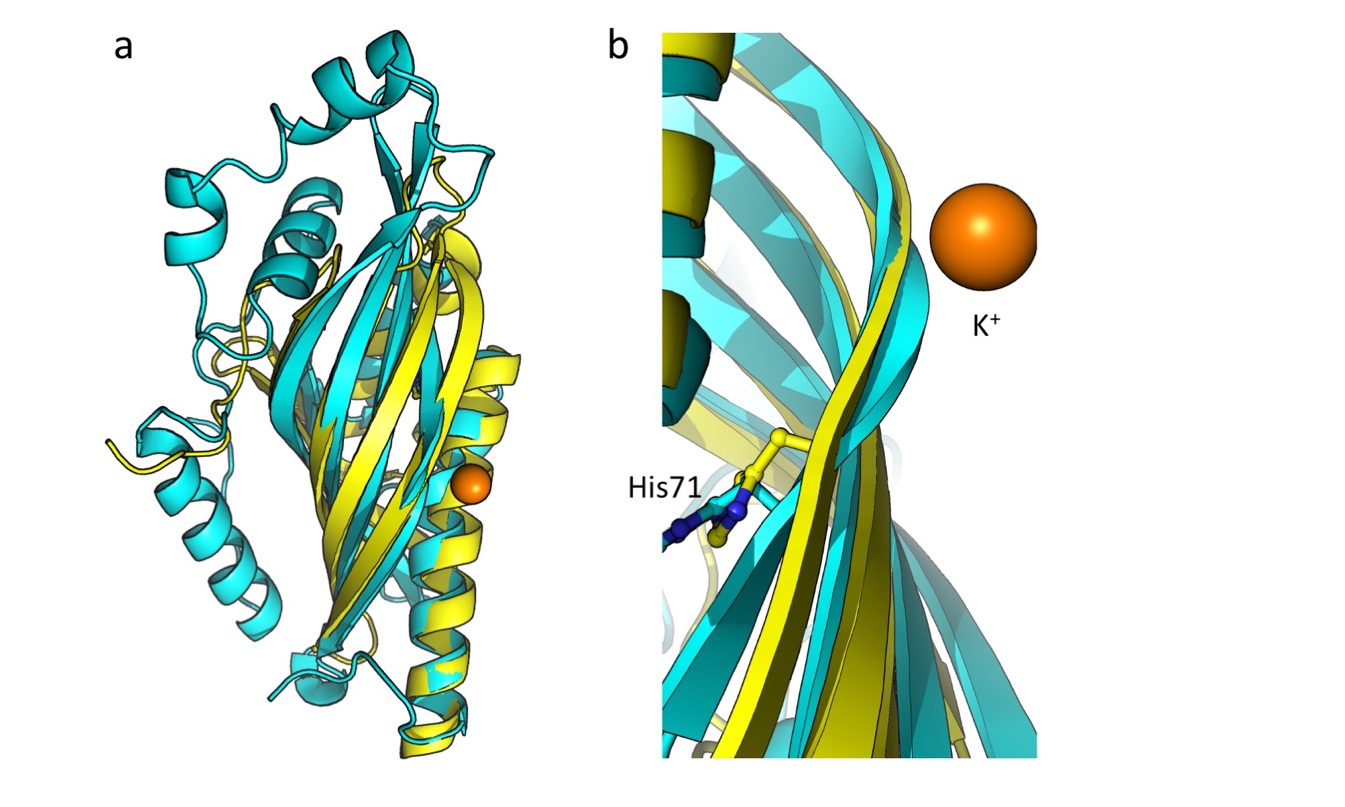


**Figure S8. Comparison between Scynth-DH and AGDC1 structures.** Structural alignment of AGDC1 (cyan) and Scyth-DH (yellow, pdb 3EF8) monomers, in global view (a) and a close-up view near the potassium binding site of AGDC1 (b). The two structures share homology in the β-core regions housing the active sites and both use a His-Asp dyad for their catalysis. In (b) His71 of Scyt-DH and His86 of AGDC1 are shown in ball-and-stick representation. The orange sphere displays the potassium ion belonging to AGDC1. No such ion is found in the center of Scyth-DH, which also lacks the twist in the β-strand corresponding to the β-strand β3 of AGDC1.

**
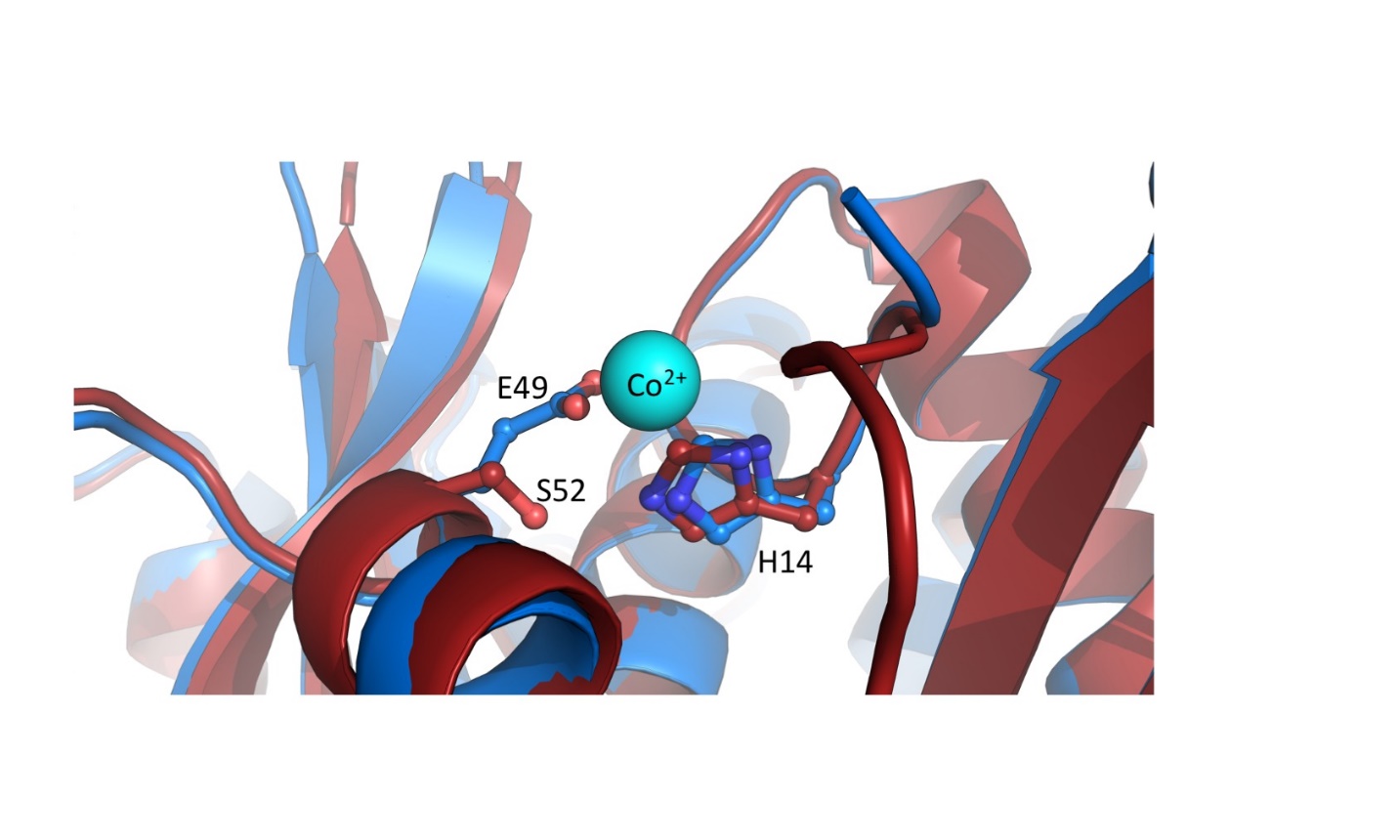
**

**Figure S9.** **Comparison between AGDC1 and PPP2 in** **the cobalt binding site.** Superposition of AGDC1-4NC/Co^2+^ and PPP2-4NC complexes showing the Co^2+^ binding site. The cobalt site from AGDC1 (blue) is absent in PPP2 (red). Although H14 is conserved in the two enzymes, E49 of AGDC1 is S52 in PPP2.

*
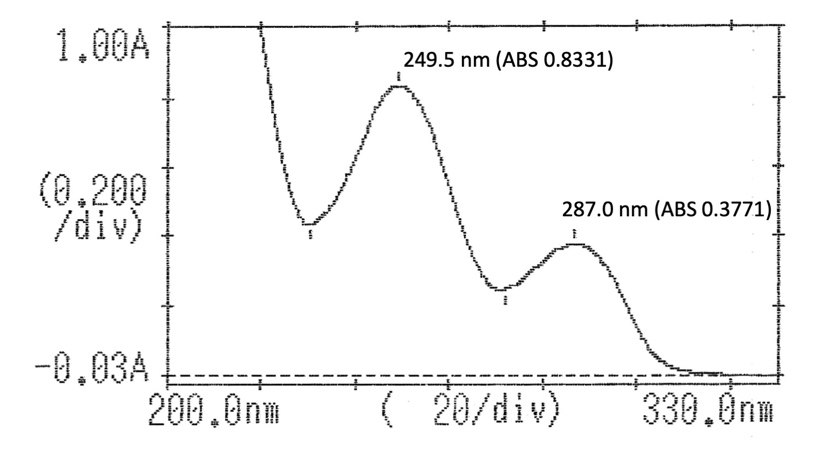
*

**Figure S10. UV spectrum scan for PCA.** Screen shot from Shimadzu UV-VIS showing the spectrum scan of ~ 0.5 mM PCA in 50 mM KPi, pH 6.5. Based on this, consumption of PCA in the enzyme assay was recorded at 250 nm.

**Figure S11. UV absorbance of GA and PCA.** GA and PCA concentrations were 0.5 mM in 50 mM KPi, pH 6.5. Measurements were performed on a Shimadzu UV-VIS spectroscope with a quartz cuvette of 2 mm pathlength. The extinction coefficients, **ε** _GA, 259 nm_ = 8635 ± 197 M^-1^cm^-1^ and **ε** _PCA, 250 nm_ = 8641 ± 237 M^-1^cm^-1^, were used for calculation of product consumption in activity assays.
